# High‐Resolution Microlens‐Assisted Tunable n‐Type Optical Doping in Monolayer MoS_2_

**DOI:** 10.1002/smll.202514203

**Published:** 2026-04-23

**Authors:** Junil Kim, Kyungjune Cho, Jieun Lee, Takhee Lee, Seungjun Chung, Hyuk‐Jun Kwon

**Affiliations:** ^1^ Department of Electrical Engineering and Computer Science Convergence Research Advanced Centre for Olfaction DGIST Daegu Republic of Korea; ^2^ Convergence Research Center for Solutions to Electromagnetic Interference in Future‐mobility (SEIF) Korea Institute of Science and Technology Seoul Republic of Korea; ^3^ Department of Physics and Astronomy, and Institute of Applied Physics Seoul National University Seoul Republic of Korea; ^4^ School of Electrical Engineering Korea University Seoul Republic of Korea

**Keywords:** 2D transition metal dichalcogenides, defect engineering, laser‐assisted microlens array processing, monolayer MoS_2_, n‐type optical doping, sulfur vacancies

## Abstract

Atomically thin two‐dimensional transition metal dichalcogenides (2D TMDCs), especially monolayer MoS_2_, have garnered considerable attention as promising materials for next‐generation transistors. However, their large surface‐to‐volume ratio renders them highly sensitive to defects, underscoring the need for selective, localized, and precise control of their defect profiles. Here, we introduce a laser‐assisted microlens array processing (LAMP) technique that enables highly localized n‐type optical doping of monolayer MoS_2_ by utilizing self‐assembled polystyrene microspheres as microlenses to focus a 532 nm continuous‐wave laser below the diffraction limit. Under low laser powers (40–60 mW), sulfur vacancies are selectively generated without inducing global thermal damage, allowing systematic control of the vacancy concentration. Spectroscopic analyses reveal electron‐donor‐like defects and tunable vacancy density. MoS_2_ transistors treated by LAMP exhibit finely tunable doping, yielding up to a 51‐fold increase in field‐effect mobility and a 37‐fold increase in carrier density, with the enhanced n‐type characteristics remaining stable for several weeks. Unlike direct laser irradiation, LAMP offers high spatial resolution, low energy consumption, and reproducible vacancy engineering while minimizing thermal damage. This complementary metal–oxide–semiconductor‐compatible strategy provides a robust post‐fabrication approach for precise electronic property tuning in two‐dimensional transition metal dichalcogenide devices.

## Graphical abstract

The laser‐assisted microlens array processing (LAMP) technique enables sub‐diffraction, localized n‐type optical doping of monolayer MoS_2_ by utilizing self‐assembled microspheres as microlenses. Controlled sulfur vacancy formation under low‐power laser irradiation allows precise tuning of the carrier concentration. This technique offers deterministic defect engineering with excellent stability while minimizing thermal damage.

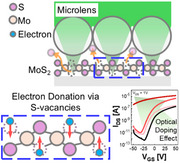

## 1 Introduction

The control of defects is a central task in semiconductor technology and materials science, yet achieving deterministic and quantitative modulation remains one of the most persistent challenges in the field. Defects govern key physical properties such as electronic structure, charge transport, and chemical reactivity, thereby directly impacting device performance and reliability [1, 2, 3, 4, 5, 6]. In semiconductors, in particular, the nature, density, and spatial distribution of defects critically influence carrier mobility, noise characteristics, and long‐term stability [7, 8, 9, 10]. Establishing strategies that enable controlled defect modulation is thus indispensable for the realization of next‐generation high‐performance, large‐scale integrated devices.

Among semiconductor materials, two‐dimensional transition metal dichalcogenides (2D TMDCs) have emerged as promising platforms due to their atomically thin geometry and superior electrostatic control [11, 12, 13, 14, 15, 16, 17]. However, their exceptionally high surface‐to‐volume ratio renders them far more sensitive to defects than their bulk counterparts, making defect control a central challenge. The type, density, and distribution of defects, as well as the presence of adsorbates, can fundamentally alter their electronic characteristics and device operation [18, 19, 20, 21, 22]. While such defects can degrade performance, they also present opportunities by enabling quantum emission, phase transitions, the creation of quantum emitters, and localized doping [23, 24, 25, 26, 27, 28]. This duality highlights the critical importance of defect engineering in 2D semiconductors, where precise control is essential to harness their beneficial potential while mitigating adverse effects.

A variety of strategies, such as thermal annealing [29, 30], electron‐beam irradiation [31, 32], plasma treatment [33, 34, 35], chemical vapor deposition (CVD) [36], and strain engineering [37, 38] have been explored to intentionally create or heal defects in TMDCs. However, these conventional defect‐engineering approaches suffer from inherent limitations, including high thermal budgets, long processing times, unintended collateral damage, and defect clustering. More critically, they lack the capability to introduce defect profiles with spatial selectivity and deterministic precision, to achieve localized doping, or to be compatible with complementary metal–oxide–semiconductor (CMOS)‐integrated processes. These intrinsic shortcomings pose a severe obstacle to the rational tuning and optimization of 2D TMDC devices.

Here, we introduce a novel laser‐assisted microlens array processing (LAMP) technique that enables spatially selective and deterministic engineering of sulfur vacancies in monolayer MoS_2_, resulting in stable n‐type optical doping and substantial enhancement of electrical performance. In this technique, optical doping refers to the modulation of electrical properties using light without any chemical dopants, enabling non‐contact and localized control of the material through optically induced defect formation that results in a stable, nonvolatile change in carrier concentration. This approach harnesses a self‐assembled microsphere array as a microlens mask to locally concentrate incident laser light into sub‐diffraction‐limited spots. Consequently, LAMP enables low‐power, site‐specific defect generation without damaging surrounding areas. Unlike conventional laser irradiation, this microlens‐assisted focusing allows precise control of vacancy density while preventing thermal degradation of the MoS_2_ lattice.

The LAMP process is cost‐effective, scalable, highly reproducible, and readily tunable through laser parameters (e.g., wavelength, power, scan rate, and spot size). Systematic spectroscopic characterization, including photoluminescence (PL), Raman spectroscopy, and X‐ray photoelectron spectroscopy (XPS), demonstrates that the sulfur vacancy density can be continuously modulated as a function of laser power. Furthermore, electrical measurements of monolayer MoS_2_ transistors confirm robust n‐type doping, substantial performance enhancement, and tunable doping levels directly governed by laser intensity, with the on‐current increased by up to 63‐fold, the field‐effect mobility enhanced by up to 51‐fold, and the carrier density raised by about 37‐fold, all while remaining stable over several weeks.

Taken together, these results establish LAMP as a powerful, spatially selective, and quantitative defect‐engineering strategy that enables fine‐tuning of electronic properties in 2D TMDCs. Beyond monolayer MoS_2_, this approach holds promise for broader application to other 2D materials and device architectures, paving the way for scalable and high‐performance 2D semiconductor logic devices.

## 2 Results and Discussion

### 2.1 Laser Processing in the LAMP Technique and the Lens Effect of Microspheres

**FIGURE 1:**
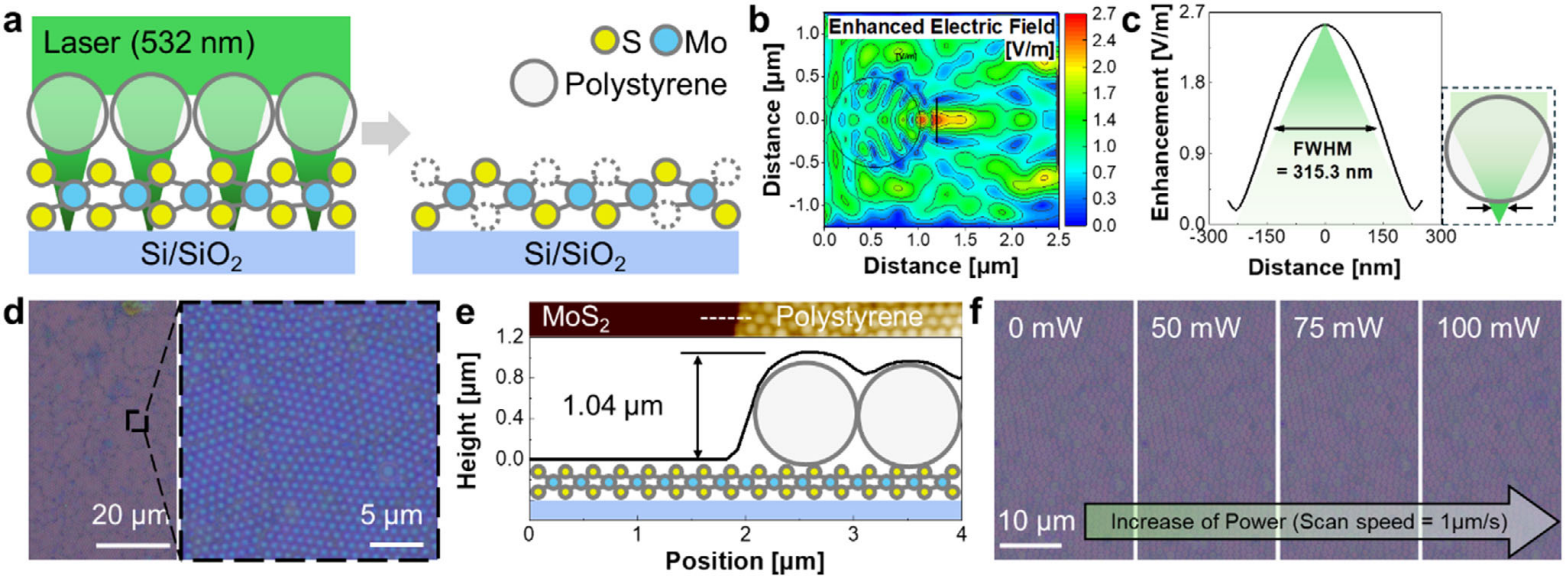
Schematic and characterization of the LAMP process. (a) Conceptual illustration of sulfur vacancy generation in monolayer MoS_2_ using LAMP with polystyrene microspheres (left: during LAMP processing, right: after removal of the microspheres). (b) COMSOL simulation of the enhanced electric field distribution transmitted through a 1.04 µm polystyrene microsphere under 532 nm laser illumination, demonstrating its microlens effect. (c) Simulated beam profile of the tightly focused light beneath the microsphere. (d) Optical microscope image of a uniform, self‐assembled monolayer of polystyrene microspheres coated on a monolayer of MoS_2_ over a large area. (e) AFM morphology measurements of coated and uncoated regions, confirming the successful deposition of 1.04 µm polystyrene microspheres. (f) Optical microscope images of the polystyrene microspheres coated on monolayer MoS_2_ after laser irradiation at a fixed scan speed of 1 µm/s and laser powers of 0, 50, 75, and 100 mW, showing that the microspheres remain undamaged within this laser power range.

Figure 1a illustrates the schematic concept of sulfur vacancy formation in monolayer MoS_2_ induced by the LAMP technique. In this study, monolayer MoS_2_ grown by CVD was transferred onto a Si/SiO_2_ substrate. Semi‐transparent polystyrene microspheres with a diameter of 1.04 µm were employed as microlenses, and a continuous‐wave (CW) laser with a wavelength of 532 nm was used as the irradiation source. The laser light is locally focused beneath the microspheres due to their lensing effect, enabling efficient defect engineering of MoS_2_ even at low laser powers [39, 40].

To validate the localized focusing effect beneath the microspheres, wave‐optical simulations were performed using COMSOL Multiphysics under the same experimental conditions: a 532 nm CW laser wavelength, 1.04 µm‐diameter polystyrene microspheres, and air as the surrounding medium. The simulation results, shown in Figure 1b,c, support that light is tightly focused beneath the microspheres, producing an approximately threefold intensity enhancement relative to the incident light. Furthermore, the full width at half‐maximum (FWHM) of the focused beam was calculated to be ∼315.3 nm. This localized optical concentration enables defect generation at incident laser powers nearly an order of magnitude lower than those typically required for conventional laser irradiation. These findings demonstrate that the LAMP technique enables highly localized and precise defect engineering at significantly lower laser intensities, highlighting the microlens role of the polystyrene microspheres.

In contrast to conventional single‐spot, diffraction‐limited laser writing, which requires serial scanning and higher local power densities to achieve periodic doping, the LAMP approach enables inherently parallel processing with improved spatial determinism, reduced inter‐dot thermal crosstalk, and significantly lower effective power density through microsphere‐assisted near‐field optical concentration. The low‐intensity, site‐specific focusing is particularly advantageous for monolayer MoS_2_, as it allows precise control of sulfur vacancy density while preventing thermal degradation of the lattice.

Figure 1d shows a uniform self‐assembled monolayer of 1.04 µm polystyrene microspheres on monolayer MoS_2_. Additional low‐ to high‐magnification scanning electron microscopy images of the self‐assembled polystyrene microspheres, confirming uniform monolayer coverage over large areas, are provided in Figure S1. The microspheres were deposited via a simple drop‐casting method using a pipette from an aqueous suspension of the spheres [41]. It should be noted that such self‐assembled microsphere patterns inherently constrain the achievable feature density, as the microsphere diameter and their proximity to the MoS_2_ surface together set an upper bound on the minimum inter‐feature spacing. The top image in Figure 1e is an atomic force microscopy (AFM) image of the MoS_2_ surface, showing both the region coated with polystyrene microspheres and the uncoated region. The bottom graph of Figure 1e shows the height profile measured along the white solid line in the AFM image, confirming that the polystyrene microspheres are uniformly distributed with a diameter of approximately 1.04 µm.

Figure 1f displays optical micrographs of the polystyrene microspheres after laser exposure at a fixed scanning speed of 1 µm/s, while the laser power was increased up to 100 mW to determine the damage threshold. No observable damage was detected below 100 mW, whereas significant degradation occurred at powers exceeding 140 mW, and micrographs for laser powers above 100 mW are provided in Figure S2. Accordingly, all laser processing was carried out at a fixed scanning speed of 1 µm/s and laser powers below 140 mW (specifically between 40 and 60 mW) to avoid damage to the microspheres. The laser spot size was approximately 5 µm, and detailed laser experimental conditions are Provided in the Experimental Section. The scanning speed and beam position were precisely controlled using an Aerotech stage system to achieve high spatial accuracy.

After the LAMP process, the polystyrene microspheres were removed by immersing the samples in toluene, which selectively dissolved the polystyrene without damaging the underlying monolayer MoS_2_ [42, 43]. Photographs of the samples before and after removal of the microspheres are shown in Figure S3.

### 2.2 Optical Characterization of Defects Induced by LAMP in Monolayer MoS_2_

**FIGURE 2:**
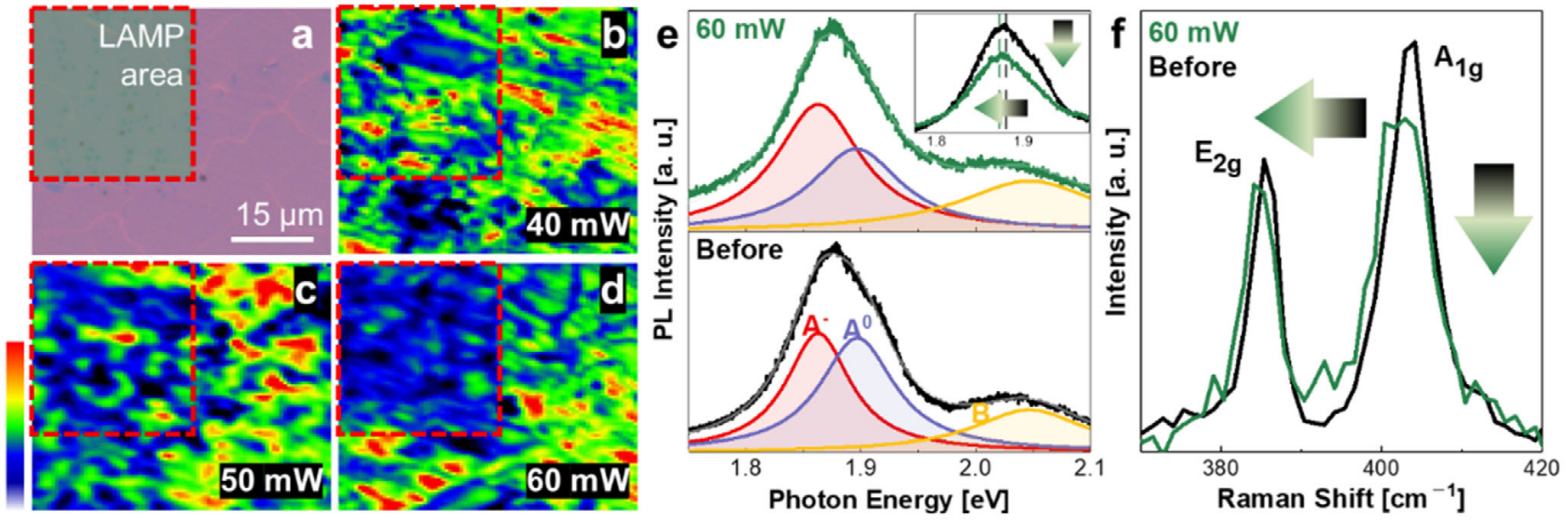
Optical characterization of monolayer MoS_2_ before and after LAMP processing at various powers. (a) Optical microscope image of monolayer MoS_2_ showing the LAMP‐processed region (red dashed box) and the unprocessed surrounding area. The LAMP process was performed over a 30 µm × 30 µm area using a zigzag pattern, and polystyrene microspheres were removed with toluene after processing. (b–d) Intensity‐integrated PL mapping images for laser powers of 40, 50, and 60 mW, respectively. The color scale corresponds to PL intensity ranging from 0 to 43 350. A progressive reduction in PL intensity is observed with increasing laser power. (e) Comparison of PL spectra before (black line) and after LAMP processing at 60 mW (green line), showing a decrease in overall intensity and a redshift in the peak position. Spectral deconvolution reveals contributions from the trion A^−^ (∼1.86 eV, red), the neutral exciton A^0^ (∼1.89 eV, purple), and the B exciton (∼2.05 eV, yellow). After LAMP treatment, the relative intensity of A^−^ increases while A^0^ decreases, indicating enhanced electron concentration due to sulfur vacancy formation. The inset shows a direct comparison of peak shifts. (f) Raman spectra of the pristine monolayer MoS_2_ and the sample processed at 60 mW. Characteristic peaks at ∼385.38 cm^−1^ (in‐plane E_2g_ mode) and ∼404.18 cm^−1^ (out‐of‐plane A_1g_ mode) exhibit reduced intensity, redshifted positions, and increased FWHM after LAMP processing. These trends confirm the generation of sulfur vacancies and associated lattice disorder.

To investigate how the optoelectronic properties of monolayer MoS_2_ evolve with laser power during the LAMP process, and to examine whether sulfur vacancies are appropriately generated, PL and Raman spectroscopy were conducted. As illustrated in Figure 2a, the LAMP process was carried out with high spatial selectivity by applying a zigzag scanning pattern (indicated by red dashed lines) over a 30 µm × 30 µm region of monolayer MoS_2_. To ensure the reliability and accuracy of signal changes in the irradiated area, the scan speed was fixed at 1 µm/s, and the process was carried out at three different positions using laser powers of 40, 50, and 60 mW in 10 mW increments. PL and Raman measurements were performed after removing the polystyrene microspheres with toluene.

Figure 2b–d shows intensity‐integrated PL mapping images corresponding to each laser power condition. A noticeable decrease in PL intensity was observed in the LAMP‐treated regions compared to the non‐irradiated ones, and this reduction became more pronounced with increasing laser power. To further analyze the peak behavior, we compared the PL spectra before and after LAMP treatment at 60 mW, as shown in Figure 2e. The inset in Figure 2e shows that the PL peak intensity decreased and the peak position shifted to lower energy after LAMP processing. This shift can be attributed to changes in electron concentration induced by an increase in sulfur vacancies [24, 44]. In monolayer MoS_2_, both PL intensity and peak position are highly sensitive to the relative population of excitons and trions. The PL spectra can be deconvoluted into contributions from the trion A^−^ near 1.86 eV (red line), the neutral exciton A^0^ near 1.89 eV (purple line), and the B exciton near 2.05 eV (yellow line) [45]. After the LAMP process, we observed a decrease in the relative intensity of the A^0^ exciton and an increase in the A^−^ trion component, indicating an increase in electron concentration. These results may be attributed to the possible formation of sulfur vacancies, which are known to act as representative electron donors with relatively low formation energy [24, 45, 46].

Defect formation was further examined by Raman spectroscopy. Figure 2f presents representative Raman spectra of the non‐irradiated sample and the sample treated with 60 mW laser power. The two characteristic peaks, corresponding to the in‐plane E_2g_ mode near 385.38 cm^−1^ and the out‐of‐plane A_1g_ mode near 404.18 cm^−1^, are separated by approximately 18.3 cm^−1^, consistent with previously reported values [47]. After the LAMP treatment at 60 mW, the intensity of both E_2g_ and A_1g_ peaks decreased by 22.7% and 34.1%, respectively. This decrease can be explained by the weakening of collective vibrations due to the formation of sulfur vacancies generated by the laser focused through the polystyrene microspheres [48]. Moreover, both E_2g_ and A_1g_ peak positions exhibited redshifts of 1.28 and 1.81 cm^−1^, respectively, which can be attributed to the reduced restoring force of the in‐plane vibrations caused by the disruption of molybdenum sulfur bonds and the generation of sulfur vacancies [49, 50]. Furthermore, we observed broadening in both Raman peaks after the LAMP process. Specifically, the FWHM of the E_2g_ and A_1g_ modes increased by 16.0% and 39.6%, respectively. Raman mapping images showing the spatial distribution of the FWHM of the E_2g_ and A_1g_ modes are provided in Figure S4. This broadening is indicative of lattice disorder and degradation of crystallinity due to defect formation [48].

The trends observed in Raman spectroscopy are consistent with the results from PL analysis, together suggesting the generation of vacancies or related defects that can act as electron donors after the LAMP process. These results suggest that the observed changes are not caused by intrinsic defects in the CVD‐grown MoS_2_ but rather by sulfur vacancies induced through the LAMP treatment. Therefore, we conclude that the LAMP process enables the effective and spatially selective generation of sulfur vacancies in monolayer MoS_2_.

### 2.3 XPS Characterization of Defects Induced by the LAMP Process in Monolayer MoS_2_

**FIGURE 3:**
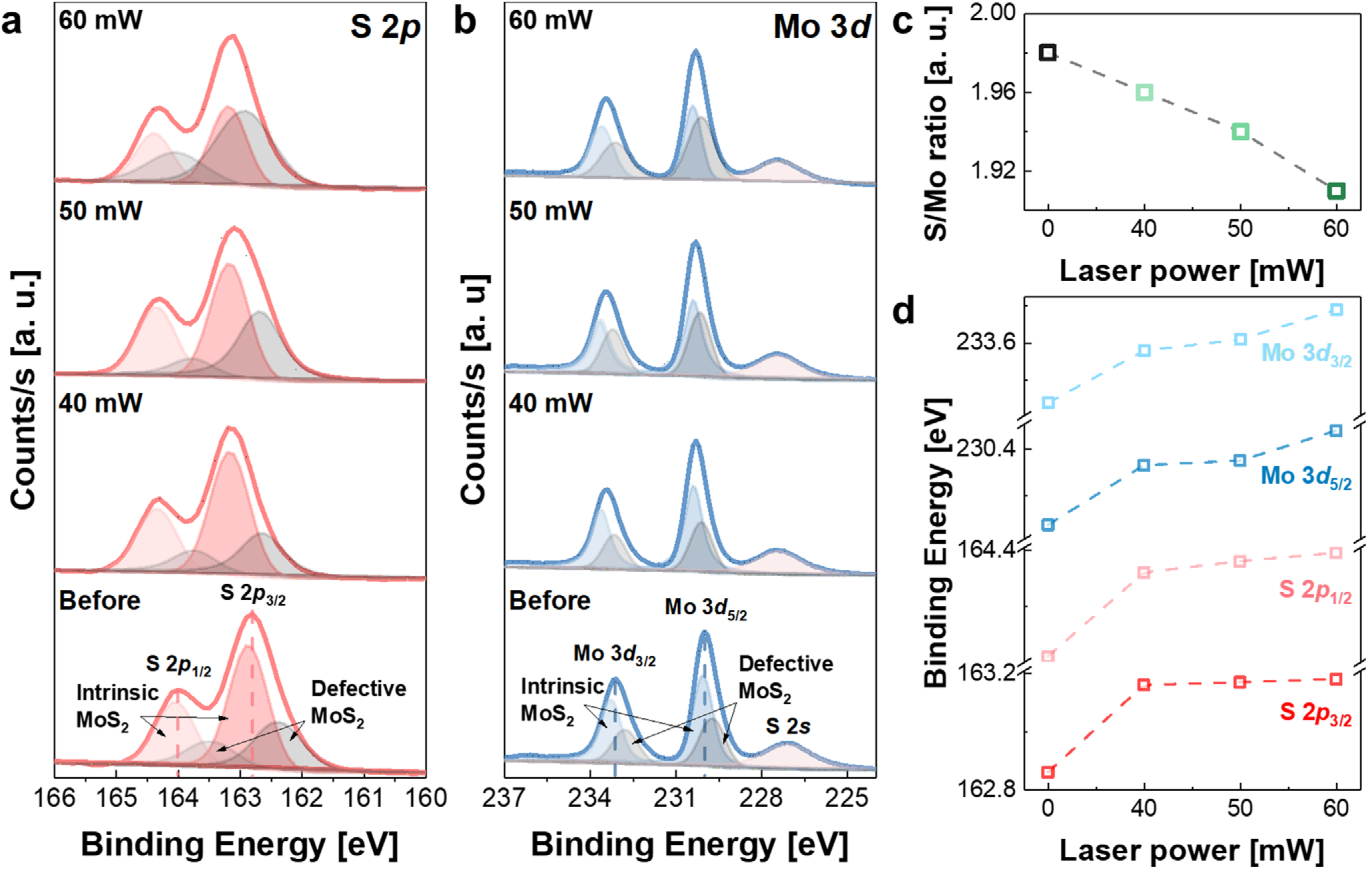
XPS analysis of monolayer MoS_2_ after LAMP processing under various laser powers (0 mW (before), 40 mW, 50 mW, 60 mW; scan speed 1 µm/s). a. S 2p spectra consist of intrinsic MoS_2_ peaks (164.0, 162.9 eV) and defective MoS_2_ peaks (163.5, 162.4 eV), with the proportion of the defective MoS_2_ component increasing as laser power rises. b. Mo 3d spectra consist of intrinsic MoS_2_ peaks (233.3, 230.1 eV), defective MoS_2_ peaks (232.8, 229.7 eV), and the S 2s peak (227.1 eV). Unlike the S 2p spectra, the proportion of the defective MoS_2_ component in the Mo 3d spectra does not show a significant increase with laser power. c. Variation of the S/Mo ratio in monolayer MoS_2_ as a function of laser power during the LAMP process. The S/Mo ratio decreases with increasing laser power, indicating the formation of sulfur vacancies. d. Shifts in the binding energies of the S 2p and Mo 3d spectra as a function of laser power, showing a gradual shift toward higher binding energies with increasing laser intensity.

XPS analysis was performed to quantitatively evaluate the defect formation and chemical bonding states in monolayer MoS_2_ as a function of laser power during the LAMP process. Figure 3a,b shows the sequential changes in the S and Mo core‐level peaks of monolayer MoS_2_ in its before state and after LAMP treatment at a fixed scan speed of 1 µm/s under varying laser powers of 40, 50, and 60 mW. To correct for surface charging effects, all binding energies were calibrated with respect to the C 1s peak of adventitious carbon at 284.8 eV. The uncorrected XPS spectra are provided in Figure S5. The XPS spectra of monolayer MoS_2_ exhibit two sets of peaks corresponding to intrinsic MoS_2_ and defective MoS_2_ generated by the LAMP process [24, 45, 51]. Both the S 2p doublets (S 2p_1/2_ and S 2p_3/2_) and Mo 3d doublets (Mo 3d_3/2_ and Mo 3d_5/2_) exhibit a downward shift of ∼0.5 eV toward lower binding energies, indicating the formation of defective MoS_2_. In addition, the S 2s peak is observed at approximately 227.1 eV.

With increasing laser power during the LAMP process, the proportion of intrinsic MoS_2_ gradually decreased, while the proportion of defective MoS_2_ progressively increased, especially evident in the S 2p peaks. By contrast, the relative proportion of intrinsic MoS_2_ and defective MoS_2_ in the Mo 3d peaks showed less significant change compared to that of the S 2p peaks. This indicates that the LAMP process predominantly induces S vacancies rather than Mo vacancies, which is consistent with the lower formation energy of S vacancies (∼3 eV) compared to Mo vacancies (∼8 eV) and aligns well with trends observed in the Raman spectroscopy and PL analysis [52]. The S/Mo ratios were determined by integrating the S 2p and Mo 3d peak areas and applying the corresponding relative sensitivity factors, as shown in Figure 3c. The calculated S/Mo ratios were 1.98 before treatment, 1.96 at 40 mW, 1.94 at 50 mW, and 1.91 at 60 mW, corresponding to estimated sulfur vacancy concentrations of approximately 1%, 2%, 3%, and 4.5%, respectively. These results demonstrate that the LAMP process provides an effective means to selectively induce sulfur vacancies in monolayer MoS_2_, while its inherently high spatial resolution enables precise control over the defect distribution.

To further assess whether other defect contributions, such as oxygen substitution or adsorption‐related species, were introduced during the LAMP process, we additionally analyzed the O 1s and C 1s core‐level spectra of monolayer MoS_2_. As shown in Figure S6, no discernible Mo–O‐related features or laser‐induced changes in oxygen‐ or carbon‐related peaks were observed across the investigated laser power range [53, 54, 55]. These results indicate that the contribution of oxygen‐containing adsorbates or carbon‐related surface contamination remains negligible and is not significantly affected by the LAMP treatment.

Figure 3d displays the shifts in the binding energies of the S 2p and Mo 3d peaks as a function of laser power during the LAMP process. Compared to MoS_2_ before treatment, all peaks shift noticeably toward higher binding energies as laser power increases. In particular, for the sample treated at 60 mW, which exhibits the highest sulfur vacancy concentration, the S 2p and Mo 3d peaks shift by approximately 0.4 eV toward higher binding energies relative to the before state. This blueshift can be attributed to the donor behavior of the sulfur vacancies generated by the LAMP process, which induces n‐type doping [23, 56]. Specifically, the increased free electron density supplied by the sulfur vacancies raises the Fermi level and subsequently shifts the core‐level peaks toward higher binding energies.

To further investigate the role of the polystyrene microspheres in the LAMP process, a control experiment was conducted by irradiating monolayer MoS_2_ under identical conditions without polystyrene microspheres. In this control experiment, laser powers of 150, 200, and 250 mW were applied, and the XPS measurements and analyses were carried out under the same conditions as those used in the LAMP process.

As shown in Figure S7, no significant changes were observed in the Mo 3d and S 2p peaks up to 200 mW, and the S/Mo ratios remained approximately 1.98 before treatment and under 150 and 200 mW, corresponding to a sulfur vacancy concentration of about 1%. However, when the laser power exceeded 200 mW (specifically at 250 mW), the S 2p core‐level spectrum showed marked broadening and a dramatic decrease in the proportion of intrinsic MoS_2_, accompanied by a substantial increase in defective MoS_2_. The S/Mo ratio dropped to approximately 0.99, corresponding to an estimated sulfur vacancy concentration of ∼49.5%.

These results are consistent with the optical simulations (Figure 1c) and collectively support the existence of a threshold energy for sulfur vacancy formation in monolayer MoS_2_. Polystyrene microspheres act as microlenses, locally enhancing the laser intensity by approximately threefold, enabling defect formation at relatively low powers (∼60 mW). In the absence of microspheres, achieving comparable defect levels requires laser powers exceeding 200 mW, which often results in excessive sulfur loss or structural degradation across the entire film [57, 58, 59]. By concentrating the laser energy into localized regions, the microspheres confine defect formation to targeted areas while minimizing lateral heat diffusion and global thermal damage, allowing controlled generation of sulfur vacancies without compromising the overall lattice integrity. This is further supported by the scanning electron microscopy image of monolayer MoS_2_ after the LAMP process, as shown in Figure S8.

### 2.4 Precise Modulation of Electrical Properties in Monolayer MoS_2_ FETs via LAMP

To evaluate whether the LAMP technique can precisely modulate the electrical properties of monolayer MoS_2_ FETs, we fabricated devices with monolayer MoS_2_ as the channel material and analyzed their electrical responses after LAMP treatment. The FETs were fabricated on heavily doped p++ Si substrates with a 300 nm‐thick SiO_2_ gate dielectric. Following the transfer of monolayer MoS_2_ onto the substrate, Ti/Au (10 nm/60 nm) source and drain electrodes were deposited via thermal evaporation. A schematic of the device structure is shown in Figure S9a. The device geometry was fixed with a channel length of 7 µm and a width of 200 µm, and multiple identical devices were fabricated for statistical analysis.

Figure S9b (left) shows an optical image of the fabricated monolayer MoS_2_ FETs, while Figure S9b (top right) depicts the same device coated with polystyrene microspheres before LAMP processing. Laser irradiation was selectively applied to the channel region at a scan speed of 1 µm/s. Following laser processing, the polystyrene microspheres were removed by immersion in toluene (Figure S9b, bottom right). No visible physical damage was observed before or after LAMP. Furthermore, as shown in Figure S10, the transfer curves before and after removing the microspheres were nearly identical, confirming that the microsphere removal process itself did not impact the electrical properties of the MoS_2_ FETs.

**FIGURE 4:**
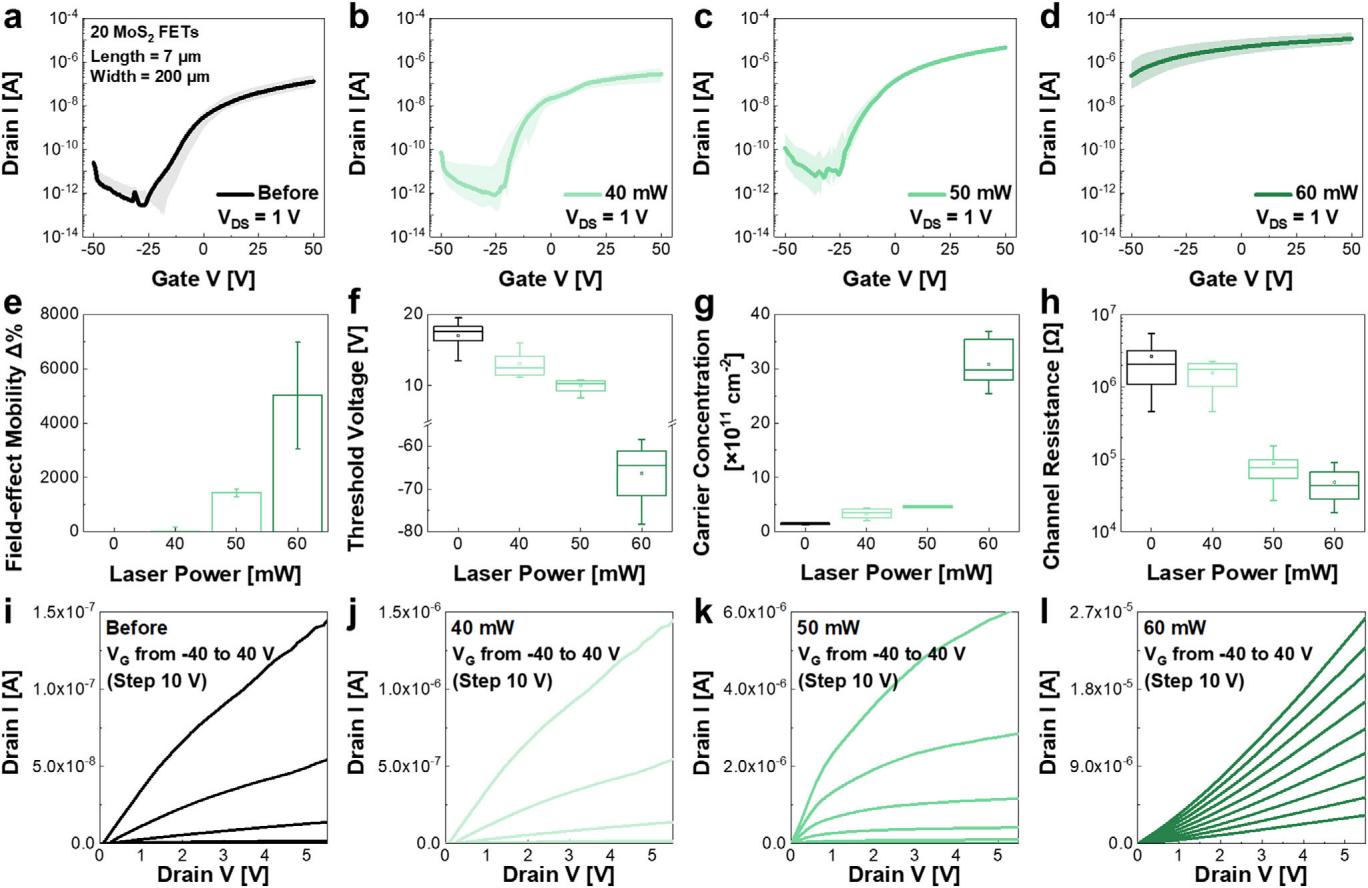
Electrical tuning of monolayer MoS_2_ FETs via n‐type doping induced by the LAMP process under various laser powers (0 mW (before), 40 mW, 50 mW, 60 mW). (a–d) Transfer characteristics of monolayer MoS_2_ FETs measured at V_DS_ = 1 V as a function of laser power during the LAMP process. To ensure reproducibility, statistical data were collected from 20 devices for each laser power. The bold curve represents the median transfer curve, while the shaded region indicates ±1 standard deviation across the devices. Specifically, (a) 0 mW (before), (b) 40 mW, (c) 50 mW, and (d) 60 mW illustrate the transfer characteristics under different laser powers. N‐type doping is evident with increasing laser power due to sulfur vacancy formation. (e–h) Changes in field‐effect mobility, threshold voltage, carrier concentration, and channel resistance as a function of laser power. (i–l) Output characteristics of monolayer MoS_2_ FETs after the LAMP process at different laser powers. V_GS_ was varied from −40 to 40 V in 10 V steps, with (i) 0 mW (before), (j) 40 mW, (k) 50 mW, and (l) 60 mW showing the corresponding output characteristics.

Figure 4a–d presents the transfer characteristics of 20 monolayer MoS_2_ FETs processed under 532 nm laser irradiation with powers of 0 mW (before), 40 mW, 50 mW, and 60 mW, using the same laser spot size of approximately 5 µm. The devices were measured at a drain voltage of 1 V. The bold line represents the median transfer curve for each condition, while the shaded region indicates ±1 standard deviation across the devices. This statistical representation is used to visualize the central trend and the device‐to‐device variation induced by the LAMP process across multiple devices. To minimize extrinsic effects from atmospheric adsorbates (e.g., O_2_, H_2_O), all devices were annealed at 140°C for 14 h under high vacuum (∼10^−7^ Torr) prior to measurements and were not exposed to ambient conditions afterward; the measurements were conducted at room temperature after annealing [60].

Increasing the laser power in the LAMP process led to a marked enhancement in device performance, demonstrating effective and controllable n‐type optical doping without introducing any extrinsic impurities. At a laser power of 60 mW, the increase in electron concentration led to a more than 63‐fold increase in the on‐current and a dramatic enhancement in the field‐effect mobility (Figure 4e), accompanied by a negative shift of the threshold voltage (Figure 4f). These enhancements strongly suggest that laser‐induced sulfur vacancies act as donor‐like defects, injecting free electrons into the MoS_2_ channel and inducing n‐type doping [61, 62, 63, 64]. The observed performance improvements highlight the advantages of the microlens‐assisted approach, demonstrating that it enables selective and highly precise tuning of electrical properties while minimizing lattice damage and preventing excessive defect clustering. Furthermore, these results are in close agreement with previously reported observations for monolayer MoS_2_, which exhibits n‐type behavior at sulfur vacancy concentrations of 1.7% and 4.7%, corresponding well to the 2%–4.5% range induced by LAMP [24].

To evaluate the long‐term stability of the LAMP process, transfer characteristics were measured immediately after the process and again after 48 days. As shown in Figure S11, the n‐type doping effect, attributed to sulfur vacancy formation, was well preserved over time, confirming the stability of the induced doping state.

To quantitatively evaluate carrier density changes, the 2D electron concentration (n_2D_) was estimated as follows [7, 65]:

(1) $$n_{2D}=\frac{I_{DS}\cdot L}{q\cdot W\cdot V_{DS}\cdot \mu_{FE}}\;$$

where q is the elementary charge, L the channel length (7 µm), W the channel width (200 µm), V_DS_ the drain‐source voltage, I_DS_ the drain current, and µ_FE_ the field‐effect mobility. As shown in Figure 4g, the average n_2D_ increased from 1.4 × 10^11^ cm^−2^ (before) to 3.4 × 10^11^ cm^−2^ (40 mW), 4.6 × 10^11^ cm^−2^ (50 mW), and 30.8 × 10^11^ cm^−2^ (60 mW), an approximately 37‐fold enhancement. These results further suggest that optical doping is feasible in MoS_2_, enabling light‐induced control of n_2D_ without any chemical dopants.

To confirm that the LAMP process selectively modified the channel region of the monolayer MoS_2_ FETs, we extracted the channel resistance as a function of laser power, as shown in Figure 4h. The methodology for extracting channel resistance is described in Figure S12 [65, 66]. The average channel resistance decreased substantially from 2625 kΩ (before) to 48 kΩ (60 mW), suggesting that the observed improvements in electrical characteristics originate from this pronounced decrease in channel resistance.

Figure 4i–l presents the output characteristics of MoS_2_ FETs treated at various laser powers with V_GS_ ranging from −40 to +40 V in 10 V steps. Consistent with the transfer characteristics, the output current increased significantly with higher laser power [24, 67, 68]. The output curves also became more linear at low V_DS_ and exhibited a reduced saturation region at high V_DS_. Notably, while devices treated at moderate laser power (e.g., 50 mW) still exhibit conventional current saturation due to effective channel pinch‐off, devices treated at higher laser power (60 mW) show a largely suppressed saturation behavior, reflecting a heavily doped channel regime. This behavior can be attributed to the increased free‐carrier density in the channel, which reduces the channel potential gradient and suppresses pinch‐off, even at high drain biases. Improved Ohmic behavior was also observed in the low‐V_DS_ region, owing to increased carrier concentration at the source and drain contacts, which mitigates band bending at the metal–semiconductor interface.

These results collectively demonstrate that the LAMP process provides highly effective and spatially selective control over sulfur vacancy density in monolayer MoS_2_, resulting in precisely tunable electronic properties. To further highlight the distinct advantages of LAMP over conventional laser irradiation, a control experiment was conducted without the polystyrene microsphere coating. The channel region was directly irradiated to evaluate laser‐induced doping without microsphere‐assisted focusing (Figure S13). While moderate irradiation produced minimal electrical changes, laser powers exceeding ∼200 mW led to rapid degradation of device performance, indicating that direct irradiation requires substantially higher energy and may induce thermal damage rather than controlled defect‐mediated doping. XPS measurements confirmed substantial thermal damage and a marked increase in defect states at 250 mW (Mo/S ratio ≈ 0.99), underscoring the critical role of the polystyrene microspheres.

These findings strongly highlight the critical role of polystyrene microspheres in LAMP processing. They enable precise and efficient energy delivery to the MoS_2_ channel while minimizing power consumption. This allows fine‐tuning of defect density with excellent spatial resolution, controllable doping concentration, and preservation of the intrinsic material properties. As a result, the microspheres are essential for achieving high‐resolution defect engineering while minimizing lattice damage and maintaining overall device integrity, as clearly evidenced by the stark contrast in electrical and structural responses compared to conventional methods. Although this study focuses on MoS_2_, the same microsphere‐assisted near‐field energy concentration mechanism is expected to be applicable to other n‐type TMDCs with similar lattice structures and chalcogen‐vacancy energetics, such as WS_2_ and MoSe_2_, with appropriate optimization of processing parameters [24, 69, 70, 71, 72].

## 3 Conclusion

In summary, we demonstrated a highly selective and controllable defect‐engineering strategy for monolayer MoS_2_ using the LAMP technique. Self‐assembled polystyrene microspheres act as optical microlenses, enabling precise, localized sulfur vacancy formation with excellent spatial resolution, low energy consumption, and preservation of intrinsic material properties.

PL and Raman spectroscopy confirmed the formation of electron‐donor‐like defects and lattice disorder, while XPS revealed tunable sulfur vacancy density through systematic shifts in S/Mo ratios and S 2p/Mo 3d binding energies. This controlled n‐type optical doping led to significant performance improvements, including ∼63‐fold increase in on‐current, ∼51‐fold enhancement in field‐effect mobility, ∼37‐fold higher carrier density, and ∼60‐fold reduction in channel resistance. The devices also exhibited stable electrical characteristics over time.

Importantly, the optical doping demonstrated in this work involves the modulation of electrical properties using light without chemical dopants, where optically induced defect formation enables localized control and results in a stable and nonvolatile change in carrier concentration.

Compared to conventional laser irradiation, LAMP provides spatially selective defect engineering with minimal lattice damage and highly reproducible control over doping, making it compatible with CMOS processes and scalable device integration. Overall, LAMP offers a versatile platform for post‐fabrication tuning of 2D TMDCs’ electronic properties, enabling high‐performance, defect‐engineered devices and paving the way for next‐generation 2D electronics.

## 4 Experimental Section

### 4.1 Preparation of CVD‐Grown Monolayer MoS_2_ Films

A heavily doped p‐type silicon substrate (p++ Si) with a 300 nm wet‐thermal SiO_2_ layer was used as the target substrate. Prior to the transfer process, the substrate was cleaned by sequential immersion in acetone and isopropyl alcohol, each heated to 80°C, for 30 min to remove surface contaminants. Commercially available CVD‐grown monolayer MoS_2_ films (10 mm × 10 mm), grown on Si/SiO_2_ substrates (SixCarbon Technology), were employed in this study. To transfer the CVD‐grown MoS_2_ onto the cleaned Si/SiO_2_ substrate, a poly(methyl methacrylate) (PMMA) layer was first spin‐coated uniformly onto the MoS_2_ film, followed by attachment of a thermal release tape on top of the PMMA layer. The sample was then immersed in a KOH solution (diluted in deionized water and heated to 60°C) to etch away the underlying SiO_2_ layer, thereby releasing the MoS_2_/PMMA/thermal tape stack from the original growth substrate. The MoS_2_ film was carefully transferred onto the freshly cleaned Si/SiO_2_ target substrate. To remove the thermal release tape, the sample was placed on a hot plate at 120°C for several seconds, allowing the adhesive to weaken and enabling easy detachment of the tape. Subsequently, the sample was annealed at 180°C for 2 min to further relax the PMMA layer, and then immersed in acetone heated to 60°C for 10 min to completely dissolve and remove the PMMA. Through this process, a clean, monolayer CVD‐grown MoS_2_ film was successfully transferred onto the new Si/SiO_2_ substrate.

### 4.2 Polystyrene Microsphere Coating and Removal

Non‐functionalized polystyrene microspheres (average diameter ∼1.04 µm, Bangs Laboratories, Inc.) were used to form a self‐assembled monolayer on monolayer CVD‐grown MoS_2_. The microspheres were diluted in deionized water and drop‐cast onto the MoS_2_ surface using a micropipette. The samples were then left to dry naturally at room temperature for several hours, leading to the formation of a uniform monolayer of microspheres. After the LAMP process, the polystyrene microspheres were selectively removed by immersing the samples in toluene for 1 h. This procedure enabled clean removal of the microspheres without damaging the underlying MoS_2_ layer.

### 4.3 Material Characterization

The morphology of polystyrene microspheres coated on monolayer MoS_2_ was characterized using an XE‐150 (Park Systems). Raman and PL measurements were carried out using an XperRAM‐S system (Nanobase, Korea) with a 532 nm excitation wavelength. XPS measurements were performed using a NEXSA G2 (Thermo Fisher Scientific), and scanning electron microscopy imaging was conducted with a Hitachi SU8020.

### 4.4 Laser Processing

To generate sulfur vacancies in monolayer MoS_2_, a LAMP technique was employed. We used a Coherent Verdi‐8 laser (Coherent, USA) operating at 532 nm, with an M2 beam quality factor < 1.1, output power stability < ±1%, beam divergence < 0.5 mrad, and a polarization ratio > 100:1 (vertical). The CW Gaussian beam was directed through a 5× objective lens (NA = 0.14), allowing it to pass through the semi‐transparent polystyrene microspheres and focus onto the underlying MoS_2_ surface, yielding a spot size of approximately 5 µm. The monolayer MoS_2_ samples were mounted on a high‐precision motorized stage (Aerotech), and scanning was performed at a speed of 1 µm/s. Laser irradiation was carried out under various power levels below 300 mW.

### 4.5 Fabrication of Monolayer CVD‐Grown MoS_2_ FETs

Monolayer CVD‐grown MoS_2_ was transferred onto a clean Si/SiO_2_ (300 nm) substrate. A negative photoresist (Merck) was spin‐coated at 3500 rpm for 40 s and soft‐baked at 110°C for 60 s. UV exposure was performed for 15 s through a square‐patterned mask (200 µm × 200 µm squares with 7 µm spacing), followed by a hard bake at 110°C for 60 s. The exposed photoresist regions were developed using AZ‐300MIF for 90 s. Subsequently, 10 nm of Ti and 50 nm of Au were deposited by thermal evaporation, and a standard lift‐off process was employed to remove unwanted metal outside the patterned regions. The resulting monolayer MoS_2_ FETs had a channel length of 7 µm and a channel width of 200 µm.

### 4.6 Electrical Characterization of Monolayer CVD‐Grown MoS_2_ FETs

The electrical characteristics of the CVD‐grown monolayer MoS_2_ FETs were measured using a computer‐controlled semiconductor parameter analyzer (Keithley 4200‐SCS). To eliminate extrinsic influences such as surface‐adsorbed impurities and to isolate defect‐induced transport behavior, the devices were annealed in high vacuum (∼10^−7^ Torr) at 140°C for approximately 14 h using a hot chuck inside a vacuum chamber, followed by natural cooling to room temperature. All electrical measurements were subsequently carried out in situ under high vacuum without exposing the devices to ambient conditions.

### 4.7 Statistical Analysis

Electrical measurements were conducted on 20 monolayer MoS_2_ FET devices fabricated under nominally identical conditions. Device‐to‐device variability was evaluated using median transfer characteristics with shaded regions representing ±1 standard deviation, together with box‐plot statistical representations. Unless otherwise specified, data are presented as statistical summaries obtained from multiple devices, with box‐plot representations used where appropriate to illustrate data distributions across devices. No additional data normalization or transformation was applied. Statistical significance testing was not performed, as the primary objective of this study was to demonstrate systematic trends induced by the LAMP process rather than to test specific statistical hypotheses. All data analysis and plotting were performed using OriginPro software.

## Conflicts of Interest

The authors declare no conflicts of interest.

## Supporting information




**Supporting File**: smll73519‐sup‐0001‐SuppMat.docx.

## Data Availability

The data that support the findings of this study are available from the corresponding author upon reasonable request.
