# Supplementary material for: High‐Resolution Microlens‐Assisted Tunable n‐Type Optical Doping in Monolayer MoS2
Source: Small. 2026 Apr 23;22(33):e14203. doi: 10.1002/smll.202514203 (PMC13262242; doi:10.1002/smll.202514203)
Supplement: Supplementary file 1 — Supporting File: smll73519‐sup‐0001‐SuppMat.docx. [file SMLL-22-e14203-s001.docx]

Supporting Information

High-Resolution Microlens-Assisted Tunable n-Type Optical Doping in Monolayer MoS_2_

Junil Kim^†^, Kyungjune Cho^†^, Jieun Lee, Takhee Lee, Seungjun Chung^*^, and Hyuk-Jun Kwon^*^

Junil Kim, Jieun Lee and Hyuk-Jun Kwon

Department of Electrical Engineering and Computer Science | Convergence Research Advanced Centre for Olfaction, DGIST, Daegu 42988, Republic of Korea

*E-mail: hj.kwon@dgist.ac.kr

Kyungjune Cho

Convergence Research Center for Solutions to Electromagnetic Interference in Future-mobility (SEIF), Korea Institute of Science and Technology, Seoul, 02792, Republic of Korea

Takhee Lee

Department of Physics and Astronomy, and Institute of Applied Physics, Seoul National University, Seoul 08826, Republic of Korea

Seungjun Chung

School of Electrical Engineering, Korea University, Seoul 02841, Republic of Korea

*E-mail: seungjun@korea.ac.kr

^†^ These authors contributed equally: Junil Kim and Kyungjune Cho


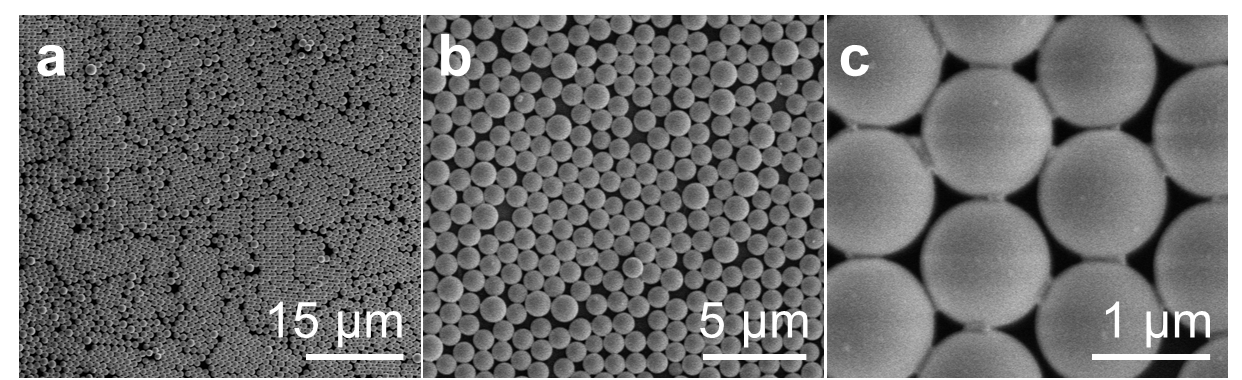


**Figures S1.** Scanning electron microscopy (SEM) images acquired at **a.** low, **b.** intermediate, and **c.** high magnifications, confirming the formation of a closely packed, self-assembled monolayer of polystyrene microspheres.


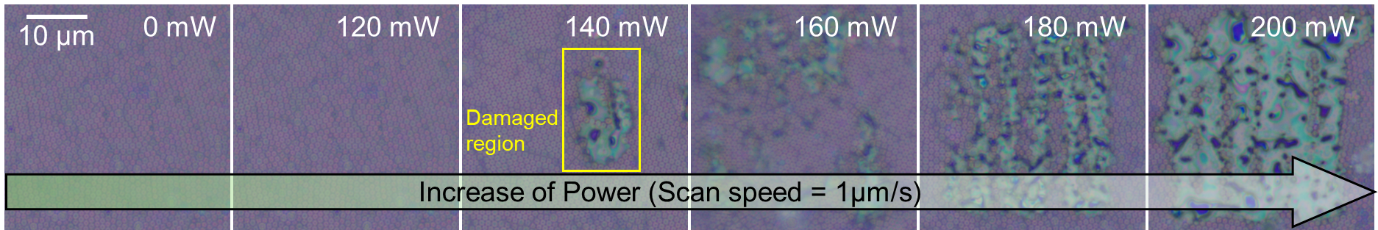


**Figure S2.** Optical microscope images of polystyrene microspheres coated on monolayer MoS_2_ after LAMP processing at various laser powers: 0 (before), 120, 140, 160, 180, and 200 mW. The laser scanning speed was fixed at 1 μm/s. Thermal degradation of the polystyrene microspheres is observed starting at a laser power of 140 mW.


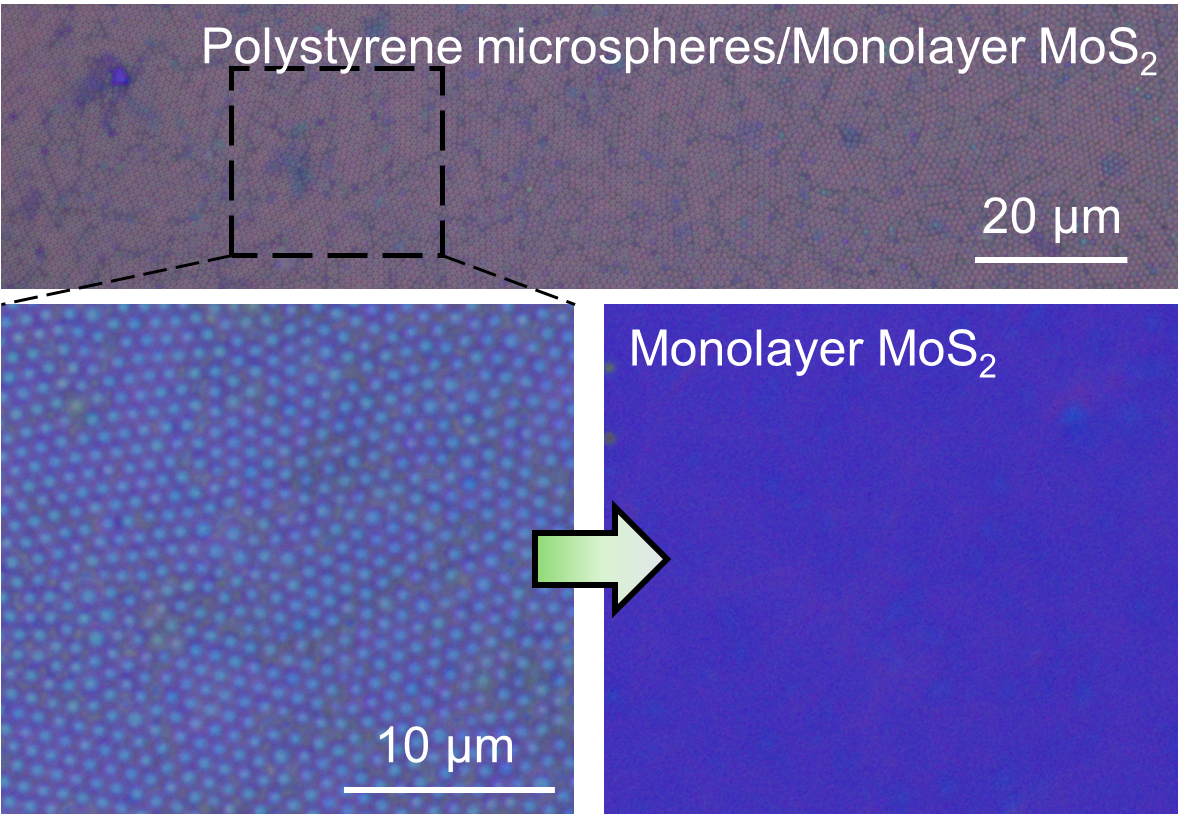


**Figure S3.** Optical microscope images showing the removal of polystyrene microspheres from the monolayer MoS_2_ surface using toluene. Polystyrene microspheres uniformly coated on monolayer MoS_2_ (Top). Magnified view of the coated region showing a close-packed polystyrene microsphere array (Bottom left). After toluene treatment, polystyrene microspheres are completely removed, revealing a clean monolayer MoS_2_ surface without observable damage (Bottom right). These results confirm that toluene effectively removes polystyrene microspheres without compromising the integrity of the underlying MoS_2_ layer.


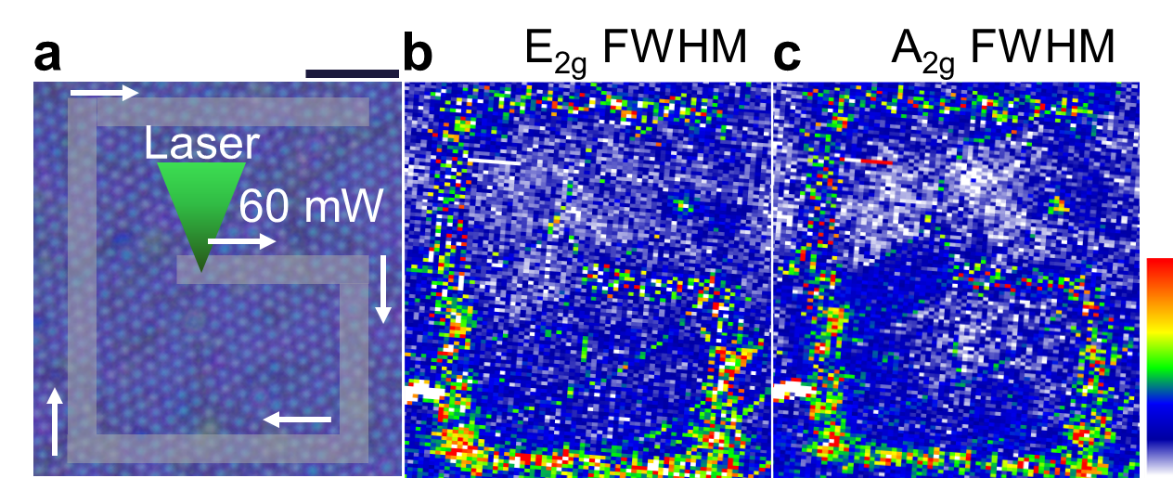


**Figure S4.** Raman mapping analysis of spatially localized defect formation induced by the LAMP process. **a.** Schematic illustration of the LAMP patterning strategy used for Raman mapping. With polystyrene microspheres coated on the surface, the LAMP process was performed by continuously scanning a 532 nm laser with a power of 60 mW in the shape of a capital letter “G”. The scale bar represents 5 µm. **b, c.** Corresponding Raman maps of the FWHM of the E_2g_ and A_1g_ modes, respectively. In both maps, a pronounced increase in peak width is observed exclusively along the laser-written “G” pattern, while the surrounding unprocessed regions remain unchanged. This spatially confined broadening directly visualizes the high degree of spatial control achievable with the LAMP process. The color scale corresponds to FWHM values ranging from 5.0 cm^−1^ (blue) to 8.9 cm^−1^ (red).


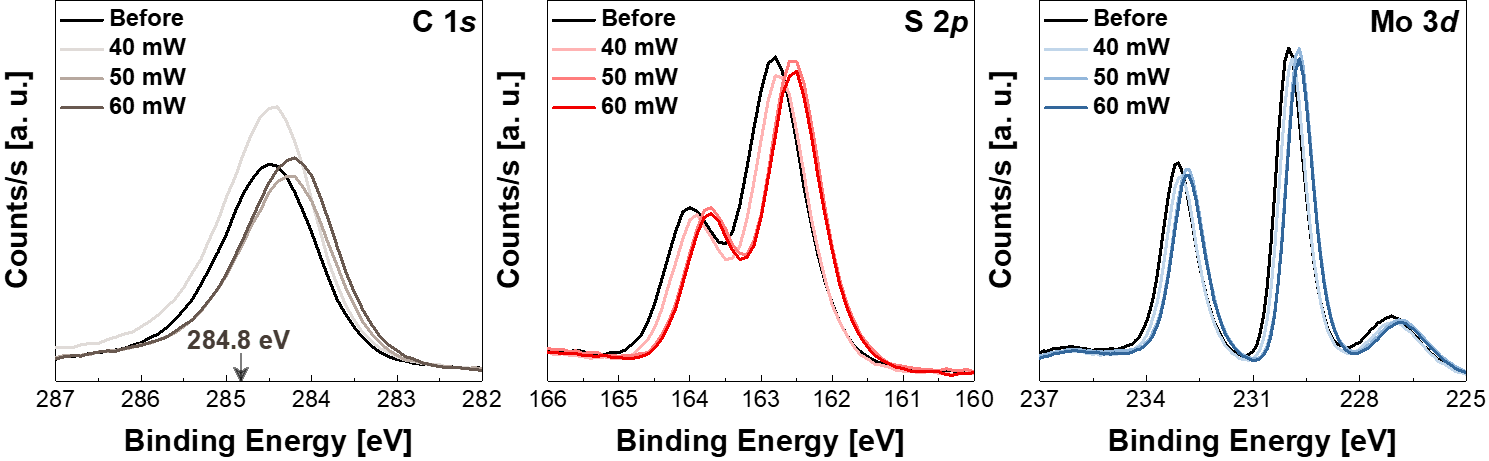


**Figure S5.** Uncorrected XPS spectra showing the effect of laser output during the LAMP process. From left to right, the spectra correspond to C 1s, S 2p, and Mo 3d regions.


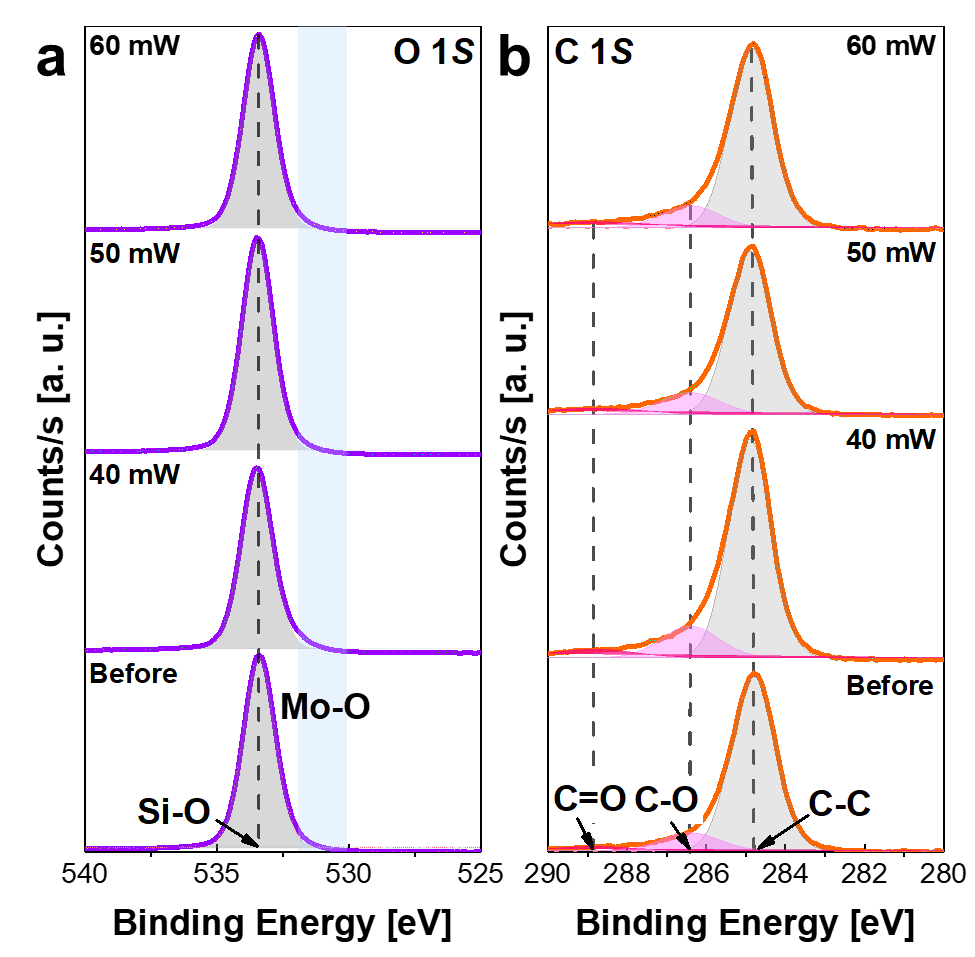


**Figure S6.** XPS analysis of monolayer MoS_2_ after the LAMP process performed at different laser powers (0 (before), 40, 50, and 60 mW) with a scan speed of 1 μm·s^−1^. **a.** O 1s spectra, in which only the Si–O-related peak at ~533.3 eV, originating from the SiO_2_/Si substrate, is observed. No detectable Mo–O (MoO_X_) component appears near ~531.4 eV (light-blue shaded region), even at higher laser powers. The minimal variation in the O 1s spectra indicates the absence of measurable laser-induced oxidation of MoS_2_ within the experimental resolution. **b.** C 1s spectra, deconvoluted into components at 284.7 eV (C–C), 286.4 eV (C–O), and 288.8 eV (C=O). No significant change in peak position or relative intensity is observed as a function of laser power, indicating negligible contributions from oxygen-containing adsorbates or carbon-related surface contamination after the LAMP process.

**Figure S6a** shows the O 1s XPS spectra of monolayer MoS_2_ measured before and after the LAMP process under various laser powers. In all cases, a single peak centered at ~533.3 eV is observed, which is attributed to Si–O bonding originating from the underlying SiO_2_/Si substrate. No discernible peak is detected in the ~531.4 eV region, where Mo–O bonding associated with MoO_X_ species is typically expected, indicating the absence of significant oxidation of MoS_2_ induced by the LAMP process.

**Figure S6b** presents the corresponding C 1s spectra of monolayer MoS_2_. The C 1s peak can be deconvoluted into three components located at 284.7 eV (C–C), 286.4 eV (C–O), and 288.8 eV (C=O). The C–C component remains dominant for all samples, and no noticeable increase in the oxygen-related C–O or C=O components is observed with increasing laser power. These results indicate that oxygen-containing adsorbates or carbon-related surface contamination are negligible and are not significantly affected by the LAMP treatment.


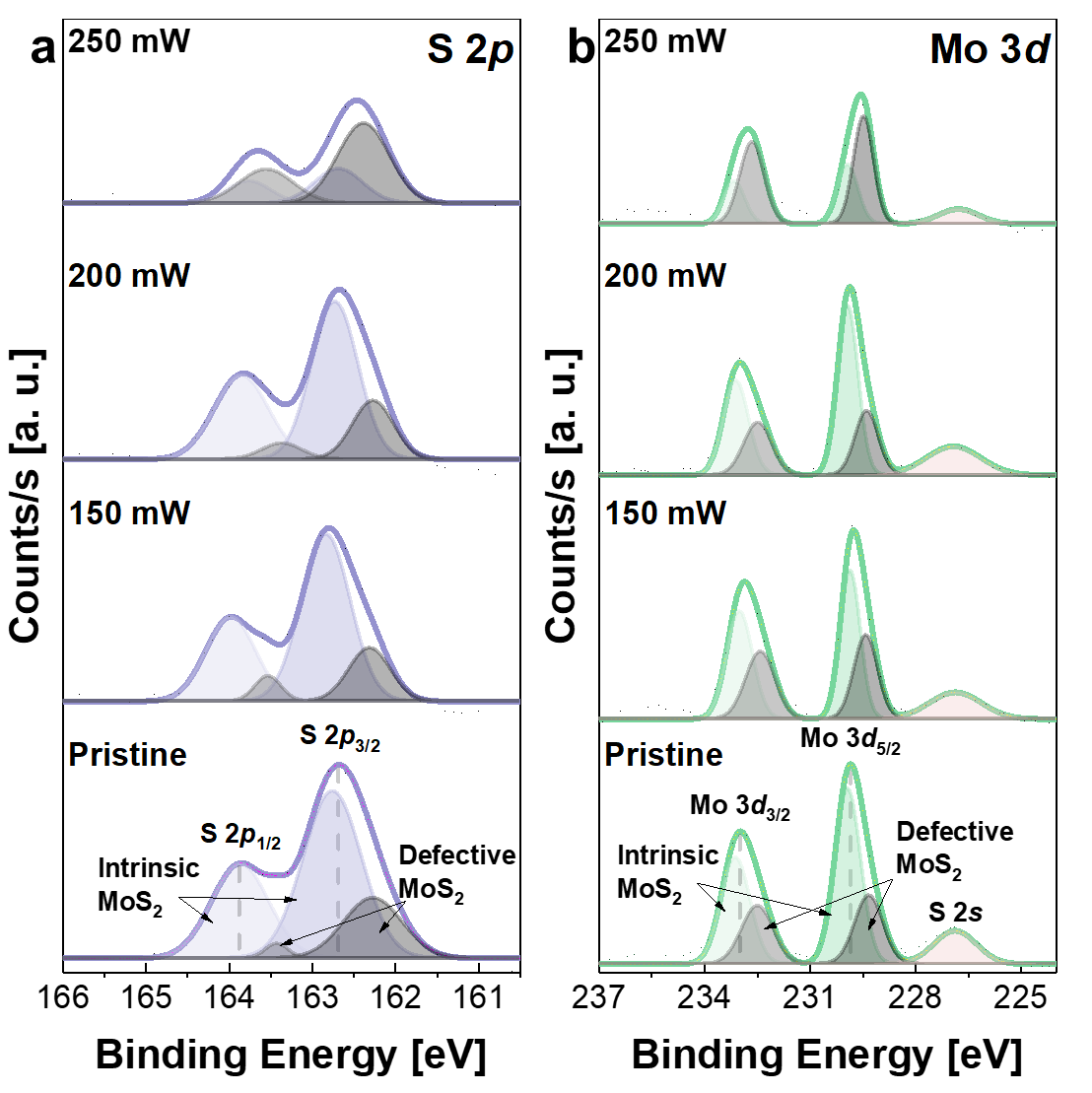


**Figure S7.** XPS analysis of monolayer MoS_2_ directly irradiated by laser without polystyrene microsphere coating. The laser scan speed was fixed at 1 μm/s, and the applied laser powers were 0 mW (before), 150 mW, 200 mW, and 250 mW. **a.** The S 2p spectra consist of intrinsic MoS_2_ peaks (~163.9 eV, ~162.8 eV) and defective MoS_2_ peaks (~163.5 eV, ~162.3 eV). Up to 200 mW, both the positions and intensities of the intrinsic and defective peaks remain nearly unchanged. However, at 250 mW, the defective MoS_2_ component increases significantly, and the S 2p peaks shift toward lower binding energies. **b.** The Mo 3d spectra consist of intrinsic MoS_2_ peaks (~233.2 eV, ~230.1 eV), defective MoS_2_ peaks (~232.6 eV, ~229.5 eV), and the S 2s peak (226.9 eV). As with the S 2p spectra, little change is observed up to 200 mW, but a significant increase in the defective MoS_2_ component is evident at 250 mW.

**
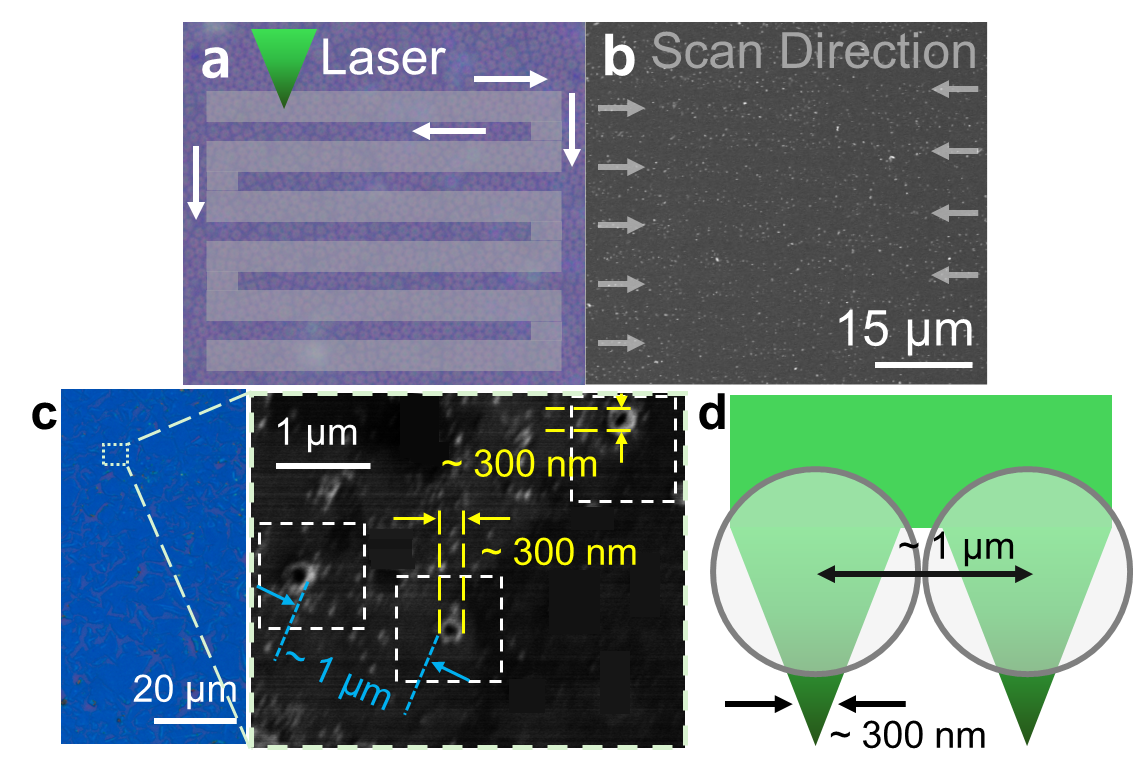
**

**Figure S8.** SEM observation of laser-induced pores in monolayer MoS_2_ after the LAMP process. **a.** Schematic illustration of the LAMP process used to visualize spatially patterned features. Laser irradiation was performed in a zigzag scanning pattern on monolayer MoS_2_ coated with a self-assembled monolayer of polystyrene microspheres. After laser processing, the microspheres were removed by toluene treatment, and the resulting MoS_2_ surface was examined by SEM. **b.** SEM image of the MoS_2_ surface after the LAMP process, showing a series of dot-like patterned features selectively formed along the laser scanning path. The periodic arrangement of these features reflects the underlying microsphere-assisted irradiation geometry. **c.** Left: Optical microscope image of monolayer MoS_2_ after the LAMP process using a laser power of 60 mW, followed by removal of the polystyrene microspheres. Right: Corresponding SEM image of the green dashed region in the optical image. Pore-like features with darker contrast relative to the surrounding area (highlighted by white dashed squares) are observed. The pores have an average diameter of approximately 300 nm (yellow dashed circles) and are spaced at intervals of ~1 μm (cyan dashed lines). **d.** Schematic illustration of the polystyrene microsphere diameter (~1 μm) and the focal width (full width at half maximum, FWHM ~300 nm) generated by the microlens effect. The ~1 μm pore spacing observed in the SEM image corresponds to the microsphere diameter, while the ~300 nm pore size reflects the microlens focal spot size, consistent with the COMSOL simulations shown in Figures 1b and 1c (FWHM = 315.3 nm).

These pores are likely the result of sulfur vacancy formation induced by the LAMP process. Regions with a high density of defects exhibit dark contrast in Scanning electron microscopy (SEM) images, due to alterations in electron absorption and emission characteristics. This confirms that defect sites are generated selectively in spatially confined areas, corresponding to the microlens-patterned regions exposed to local laser irradiation.


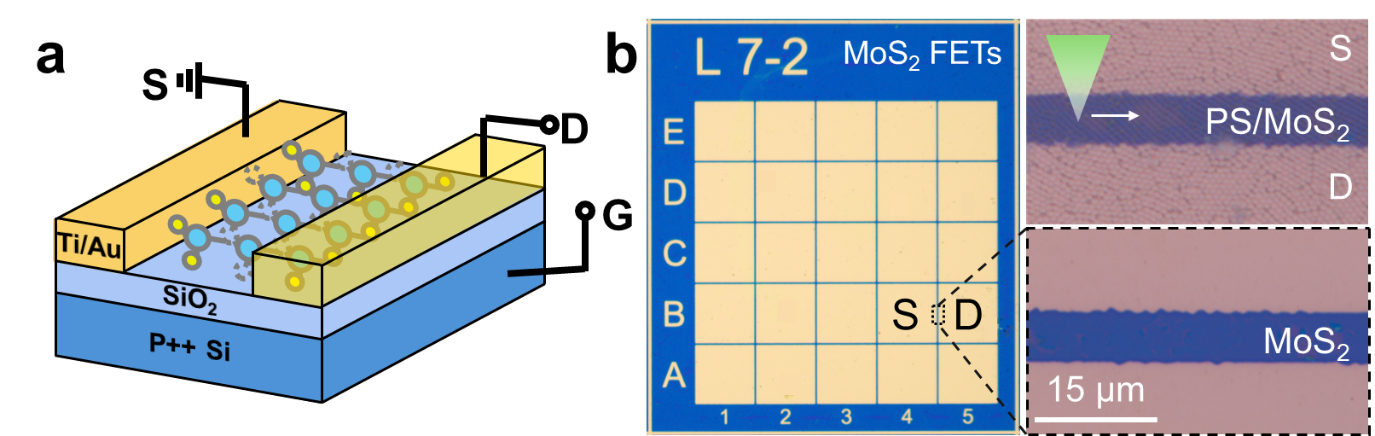


**Figure S9.** Device structure and optical microscope images of monolayer MoS_2_ FETs used in the LAMP process. **a.** Schematic illustration of a back-gated monolayer MoS_2_ FET with Ti/Au source and drain electrodes on a Si/SiO_2_ substrate. **b.** Representative optical image of monolayer MoS_2_ FETs on the chip (left). Optical microscope image of a device with polystyrene microspheres coated over the channel and contact regions (top right). The polystyrene microspheres were selectively irradiated only over the channel area to enable localized LAMP processing. Optical image of the same device after toluene treatment shows the complete removal of polystyrene microspheres from the channel region (bottom right).

**Figure S10.** Comparison of representative transfer curves from four monolayer MoS_2_ FETs before and after toluene treatment. The negligible change in electrical characteristics after toluene exposure confirms that toluene effectively removes polystyrene microspheres without inducing any material or electrical property alterations in the monolayer MoS_2_, such as additional defect formation or structural deformation.

**Figure S11.** Transfer characteristics of monolayer MoS_2_ FETs measured before LAMP processing, immediately after processing (0 days), and 48 days post-processing to evaluate the stability of optical n-type doping. The LAMP process was performed using a laser power of 60 mW. The results confirm that the n-type doping effect remains stable over several weeks.


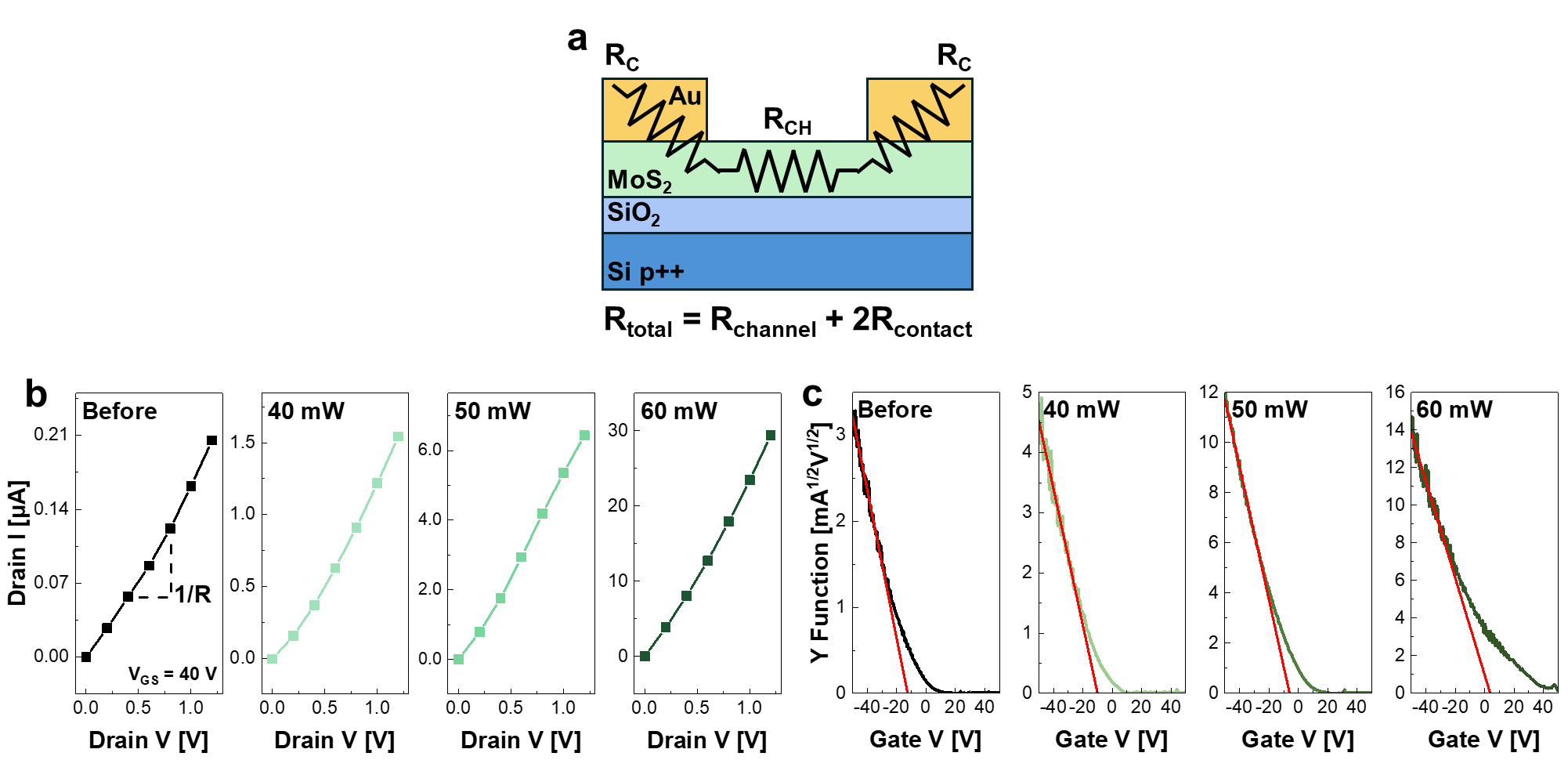


**Figure S12.** Extraction of contact resistance (R_C_) and channel resistance (R_CH_). **a.** Schematic illustration of the relationship between the total resistance (R_total_​), R_CH_, and R_C_​ in monolayer MoS_2_ FETs. The R_CH_ is calculated as R_CH_ = R_total_ − 2R_C_​. **b.** Output characteristics of monolayer MoS_2_ FETs at V_GS_ = 40 V in the low V_DS_​ regime for various laser powers during the LAMP process. The R_total_ is extracted from the inverse of the slope of the I_DS_$-$V_DS_​ curve. **c.** Y-function analysis for different laser powers, showing the relationship between the Y-function and V_GS_​. The red solid lines indicate linear fits in the linear region, from which the 2R_C_​ is extracted.

To indirectly evaluate the change in channel resistance (R_CH_) of monolayer MoS_2_ FETs subjected to varying laser power during the LAMP process, we employed the Y-function method for systematic extraction. As depicted in **Figure S12a**, the total device resistance can be described by the equation:

R_total_ = R_CH_ + 2R_C_

where R_C_​ is the contact resistance at the source/drain interfaces. Accordingly, the channel resistance R_CH_​ was calculated by subtracting the extracted contact resistance from the total measured resistance. The total resistance R_total_​ was extracted from the output characteristics at V_GS_ = 40 V, specifically in the low V_DS_ region (0 to 1.2 V), as shown in Figure S9b. To determine the contact resistance, we analyzed the Y-function versus V_GS_​ plot presented in Figure S9c. The Y-function of the transistor is defined as:

Y = $\frac{I_{DS}}{\sqrt{g_{m}}}$ = $\sqrt{\frac{W}{L}C_{OX}\mu_{0}V_{DS}}\times(V_{GS}-V_{TH})$

where W and L are the channel width and length, C_OX_ is the gate oxide capacitance, V_GS_ is the gate-source voltage, V_TH_ is the threshold voltage, V_DS_ is the drain-source voltage, $\mu_{0}$ is the intrinsic carrier mobility. The slope of the linear regime in **Figure S12c** (highlighted by the red line) corresponds to the parameter $\sqrt{\frac{W}{L}C_{OX}\mu_{0}V_{DS}}$,which is subsequently used to extract 2R_C_.

Finally, R_CH_​ was calculated by subtracting 2R_C_​ from R_total_​, as described above. The extracted R_CH_​ values for different laser power conditions are summarized in **Figure 4h**.


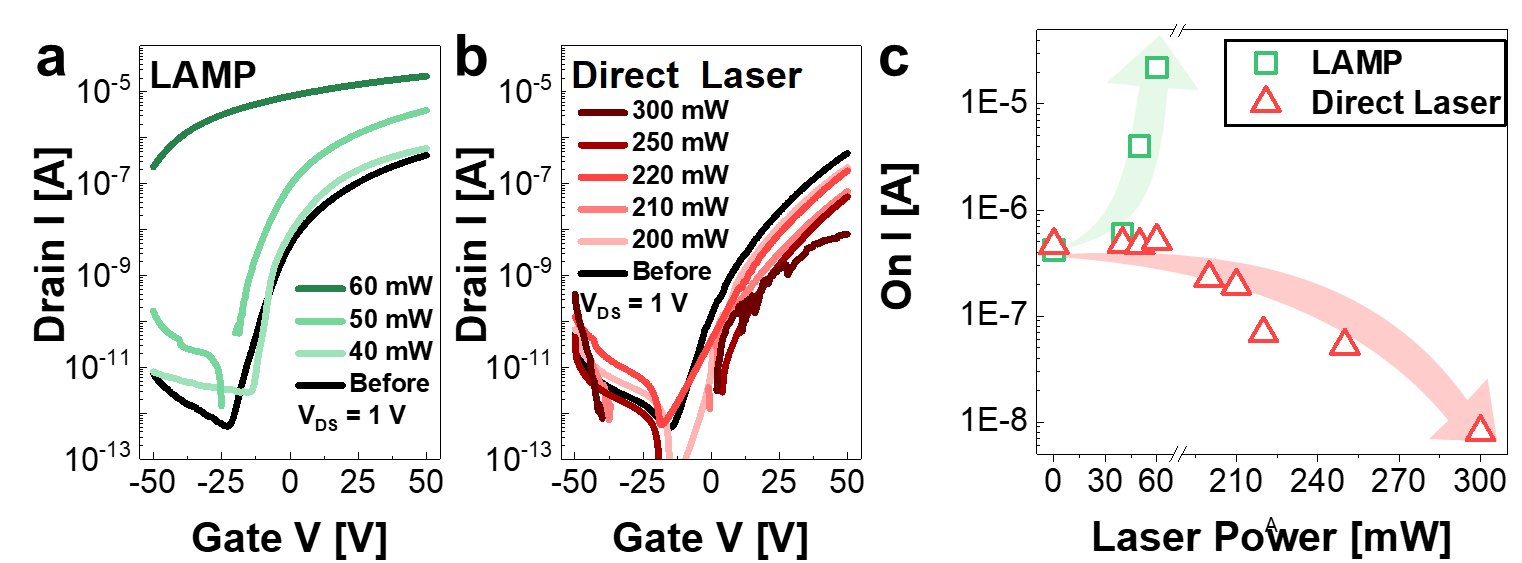


**Figure S13.** Side-by-side electrical comparison of monolayer MoS_2_ FETs processed by the LAMP technique and conventional direct laser irradiation. **a.** Transfer characteristics (V_DS_ = 1 V) of monolayer MoS_2_ FETs subjected to the LAMP process at laser powers ranging from 0 to 60 mW, showing systematic modulation of the electrical behavior with increasing laser power. **b.** Transfer characteristics (V_DS_ = 1 V) of monolayer MoS_2_ FETs processed by direct laser irradiation without microspheres at laser powers ranging from 0 to 300 mW. A pronounced degradation of electrical performance is observed at laser powers exceeding ~200 mW. **c.** Side-by-side comparison of the on-state current (V_DS_ = 1 V) of monolayer MoS_2_ FETs as a function of laser power for the LAMP process and direct laser irradiation. While LAMP induces a significant increase in on-current at relatively low laser powers, direct laser irradiation fails to produce a comparable doping effect, likely due to thermal accumulation and insufficient spatial confinement.

To quantitatively compare the LAMP process with a conventional laser-based approach, a side-by-side device-level study was conducted using direct laser irradiation without microspheres as a reference. As shown in **Figure S13**, the LAMP-processed devices exhibit a pronounced and controllable n-type doping effect at relatively low laser powers of 40–60 mW, consistent with microsphere-assisted near-field optical enhancement. In contrast, devices subjected to direct laser irradiation show little to no change in electrical characteristics up to ~200 mW, beyond which the device performance rapidly degrades.

This degradation is attributed to excessive heat accumulation and the lack of spatial confinement during direct laser irradiation, which hinders controlled defect formation and instead leads to irreversible material damage. The comparison of on-current clearly demonstrates that, while LAMP enables efficient modulation of the electrical properties at low power, direct laser irradiation requires substantially higher energy input and fails to achieve comparable doping levels before thermal deterioration occurs.

These results indicate that the laser power density required to induce n-type doping via direct irradiation approaches the regime of irreversible thermal degradation, whereas the LAMP technique enables efficient, low-power, and spatially confined doping through near-field optical concentration and parallelized microsphere-assisted processing. This side-by-side comparison highlights the clear advantage of LAMP over conventional direct laser approaches in terms of controllability, thermal management, and doping efficiency.
